# Harnessing the Rhizosphere of the Halophyte Grass *Aeluropus littoralis* for Halophilic Plant-Growth-Promoting Fungi and Evaluation of Their Biostimulant Activities

**DOI:** 10.3390/plants10040784

**Published:** 2021-04-16

**Authors:** Mohamed Tarroum, Walid Ben Romdhane, Ahmed Abdelrahim Mohamed Ali, Fahad Al-Qurainy, Abdullah Al-Doss, Lotfi Fki, Afif Hassairi

**Affiliations:** 1 Laboratory of Plant Biotechnology Applied to Crop Improvement, Faculty of Sciences of Sfax, University of Sfax, B.P. 802, Sfax 3038, Tunisia; mtarroum@ksu.edu.sa (M.T.); lotfifki@yahoo.fr (L.F.); 2 Department of Botany and Microbiology, College of Science, King Saud University, Riyadh 11451, Saudi Arabia; fahad_alqurainy@yahoo.com; 3 Plant Production Department, College of Food and Agricultural Sciences, King Saud University, P.O. Box 2460, Riyadh 11451, Saudi Arabia; walid.brm3@gmail.com (W.B.R.); asaif@ksu.edu.sa (A.A.M.A.); aaldoss@ksu.edu.sa (A.A.-D.); 4 Centre of Biotechnology of Sfax, University of Sfax, B.P. 1177, Sfax 3018, Tunisia

**Keywords:** plant growth promoting fungi, biostimulants, indole acetic acid, brassinosteroid, nitrogen metabolism

## Abstract

Hydroponic systems have gained interest and are increasingly used in hot and dry desert areas. Numbers of benefits are offered by hydroponic systems such as the ability to save water, enhance nutrients use efficiency, easy environmental control, and prevention of soil-borne diseases. However, the high consumption of chemical fertilizers for nutrient solution and the sensitivity of closed hydroponic systems to salinity are issues that need solutions. Thus, the main goal of our research activities is to isolate plant growth promoting fungi in order to develop sustainable hydroponic systems. We are working on isolating and testing the possibility to incorporate the cell-free filtrate (CFF) of plant growth promoting fungi (PGPF) in the composition of the nutrient solution. In this work, we isolated six strains of PGPF from the rhizosphere of the halophyte grass *Aeluropus littoralis*. Phylogenetic analyses of DNA sequences amplified by ITS1 and ITS4 primers identified the isolated fungi as: *Byssochlamys spectabilis*, *Chaetomium globosum*, *Cephalotheca foveolata*, *Penicillium melinii*, *Alternaria tenuissima*, and *Nigrospora chinensis*. The promoting of vigor in tobacco seedlings was used as criteria to evaluate the biostimulant activity of these fungi by adding either their mycelia (DE: direct effect) or their cell-free filtrates (CFF: indirect effect) to the plant-growth media. The best significant growth stimulation was obtained with plants treated by *B. spectabilis*. However, only the CFFs of *Byssochlamys spectabilis* (A5.1) and *Penicillium melinii* (A8) when added at a dilution factor of 1/50 to half-strength nutritive solution (0.5NS) resulted in significant improvement of all assessed growth parameters. Indeed, the A5.1CFF and A8CFF in 0.5NS induced a significant better increase in the biomass production when compared to NS or 0.5NS alone. All fungi produced indole acetic acid in the CFFs, which could be one of the key factors explaining their biostimulant activities. Furthermore, six genes involved in nitrogen-metabolism (*NR1* and *NRT1*), auxin biosynthesis (*Tryp1* and *YUCCA6-like*), and brassinosteroid biosynthesis (*DET2* and *DWF4*) were shown to be induced in roots or leaves following treatment of plants with the all CFFs. This work opens up a prospect to study in deep the biostimulant activity of PGPFs and their applications to decrease the requirement of chemical fertilizers in the hydroponic growing systems.

## 1. Introduction

The world population will increase by at least 1.5 billion people over the next 50 years. Hence, the increasing crop production outputs to ensure enough food provision will become an urgent necessity [1]. The ‘green revolution’ has participated in increasing grain production. However, this is achieved by intensive irrigation and chemical fertilization, based on the use of nitrogen (N), phosphate (P), and potassium (K) [2]. Moreover, the different biotic stresses, various abiotic stresses, and nutrient stress restrict crop plants from reaching their full genetic potential and cause significant yield losses worldwide [3]. To solve these problems and improve the agriculture sustainability, many innovative technologies have been implemented. Plant biotechnology has been used to produce new varieties with increased tolerance to abiotic or biotic stresses and add nutritional value. Furthermore, during the last two decades, plant biostimulants, based on natural materials, have received considerable attention from the scientific community and commercial enterprises [4]. Biostimulants can enhance plant development and growth from germination to maturity. They also induce yield increases, improvement of crop quality, better nutrient assimilation, and enhance plant tolerance to abiotic stress [5]. The main categories of plant biostimulants were classified by du Jardin [6] as: humic and fulvic acids, protein hydrolysates, other N-containing compounds, seaweed extracts, chitosan, biopolymers, inorganic compounds, and plant growth promoting microorganisms (such as beneficial bacteria and fungi: biofertilizers). The exploitation of beneficial plant–microbe interactions offers promising and environmentally friendly strategies for implementing sustainable agriculture. It decreases the excessive use of fertilizers, especially in hydroponics [7]. Plant growth-promoting microorganisms have played a significant role in the move toward sustainable agriculture, as their application improves soil fertility, agricultural production, and food quality. Enhanced crop production through microorganism-plant interactions can be achieved directly by promoting increases in water and nutrient uptake and the production of growth regulators. Moreover, their action can occur indirectly through the production of antibiotics and cell wall lytic enzymes, which contribute to the suppression of pathogens [8]. Among the plant growth-promoting microorganisms, fungi from the rhizosphere have been known to benefit plant development. Moreover, fungi represent the largest components of the soil microbiota and soil biomass [9]. Therefore, plant growth promoting fungi (PGPF) have provided new options to researchers worldwide as an alternate strategy for minimizing the use of chemical fertilizers. This positive effect has been shown to occur through direct or indirect fungi–plant interactions, due to the improved capacity for nutrient acquisition and hormonal stimulation. The mechanisms of growth stimulation via PGPF involve the decomposition of organic matter, production of plant hormones, enhancement in mineral uptake, solubilization of inorganic phosphate, production of phytase/siderophores, and protection of plants from biotic and abiotic stresses [10,11]. PGPF have also been identified as biofertilizers, owing to their multiple qualitative and quantitative beneficial effects on plant and their positive relationship with the environment [12]. Beneficial fungi have stimulated growth promoting effects in a variety of host plants such as maize, pea, chickpea, cucumber, and rice [9,10,13]. In addition to the direct effect of mycelium on plant growth, fungal cell-free filtrates have also been shown to promote the growth. Enhanced tobacco growth was observed when the roots were exposed to fungal cell-free culture supernatants of the fungus *Isaria javanica* pf185 [14]. On the other hand, the addition of autoclaved and filter-sterilized culture filtrate of *Piriformospora indica* resulted in a significant enhancement in growth of *Linum album* plant [15]. Khan et al. [16] showed that the culture filtrate of the endophytic fungi isolated from *Solanum nigrum* leaves had the capability to promote the growth of rice plants. Moreover, the growth of cucumber [17] and maize plants [18] was significantly enhanced in response to the CFFs of *Penicillium simplicissimum* and *Piriformospora indica* respectively.

It has been reported that the dialogue between beneficial fungi and plants are associated with molecular mechanisms that span the two partners through a signaling network that involves their respective genomes [19,20]. However, the molecular mechanisms and genes responsible remain to be explored.

The hydroponics system usually reduces the nutrients and water use with substantial benefit for the environment especially if it is combined with PGPF. In this work, we isolated fungi from the rhizosphere of a halophyte grass *Aeluropus littoralis* growing in sebkha. In addition, we screened their plant-growth promotion activity and the cell-free culture filtrate (CFF) of these PGPFs were tested at 1/50 dilution factor in nutrient solution used in hydroponic growing systems in order to decrease the chemical fertilizers inputs. Finally, the expression profiles of some genes involved in plant growth were investigated.

## 2. Results

### 2.1. Characterization and Identification of Isolated Fungi

#### 2.1.1. Microscopic and Molecular Characterization

The Six isolated and purified fungi grown on PDA were identified, based on their morphology (surface texture, colony shape, growth rate, color) and microscope observations (Supplementary Figure S1).

**Figure 1:**
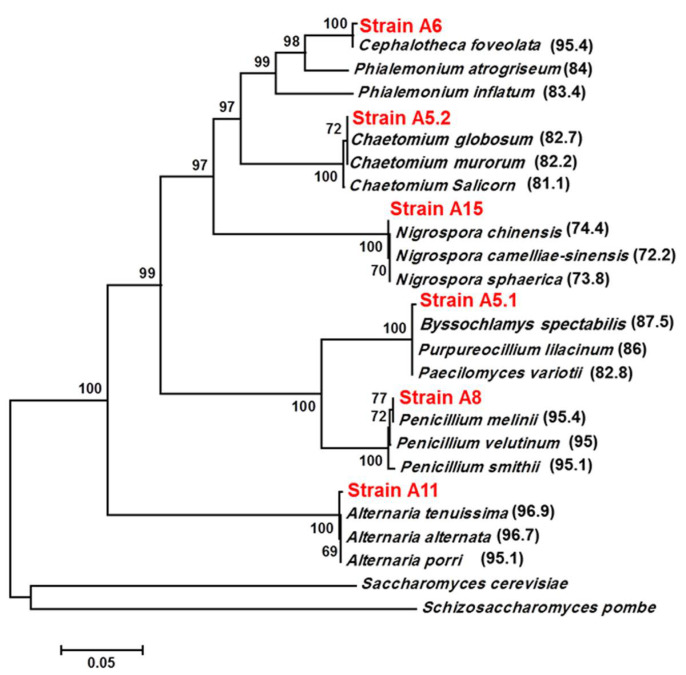
Phylogenetic tree for A5.1, A5.2, A6, A8, A11, A15 and related strains based on the ITS sequences. GenBank accession numbers of different ITS sequences used in constructing the phylogenetic tree are as follows: *Byssochlamys spectabilis* (MH512976), *Purpureocillium lilacinum* (KC157750), *Paecilomyces variotii* (AF033395), *Chaetomium globosum* (MN856296), *Chaetomium salicorn* (KR093181), *Chaetomium murorum* (KM268657), *Cephalotheca foveolata* (KJ573100), *Phialemonium atrogriseum* (AB540569), *Phialemonium inflatum* (MH859036), *Penicillium melinii* (MH858072), *Penicillium velutinum* (MH855309), *Penicillium smithii* (MH862076), *Alternaria tenuissima* (KM921667), *Alternaria alternata* (MK332247), *Alternaria porri* (JF422730), *Nigrospora chinensis* (KX985947), *Nigrospora camelliae-sinensis* (KX986015), *Nigrospora sphaerica* (JN198501), *Saccharomyces cerevisiae* (MG775707), and *Schizosaccharomyces pombe* (MH595429). The phylogenetic tree was constructed by the neighbor-joining method with 1000 replicates using MEGA7 software. Numbers in parentheses are the percentages of identity between the ITS sequences (%).

The ITS regions of the six strains were amplified using ITS1 and ITS4 primers and the bands ranging from 550 to 600 bp (Supplementary Figure S2) were cloned in a pGEMT-easy vector for sequencing. Using the BLASTN tool in the NCBI GenBank, we showed that the six isolated strains belong to the phylum Ascomycota. Moreover, phylogenetic analysis using the neighbor-joining method indicated that the isolates were presumed to be *Byssochlamys spectabilis* (A5.1), *Chaetomium globosum* (A5.2), *Cephalotheca foveolata* (A6), *Penicillium melinii* (A8), *Alternaria tenuissima* (A11), and *Nigrospora chinensis* (A15), with similarities of 98.34%, 99.82%, 99.63%, 99.82%, 99.48%, and 99.88%, respectively (Figure 1). Furthermore, the strains belonged to four different orders, *Eurotiales* (A5.1 and A8), *Sordariales* (A5.2, A6), *Pleosporales* (A11), and *Trichosphaeriales* (A15).

#### 2.1.2. Effect of Temperature and NaCl on Growth

**Figure 2:**
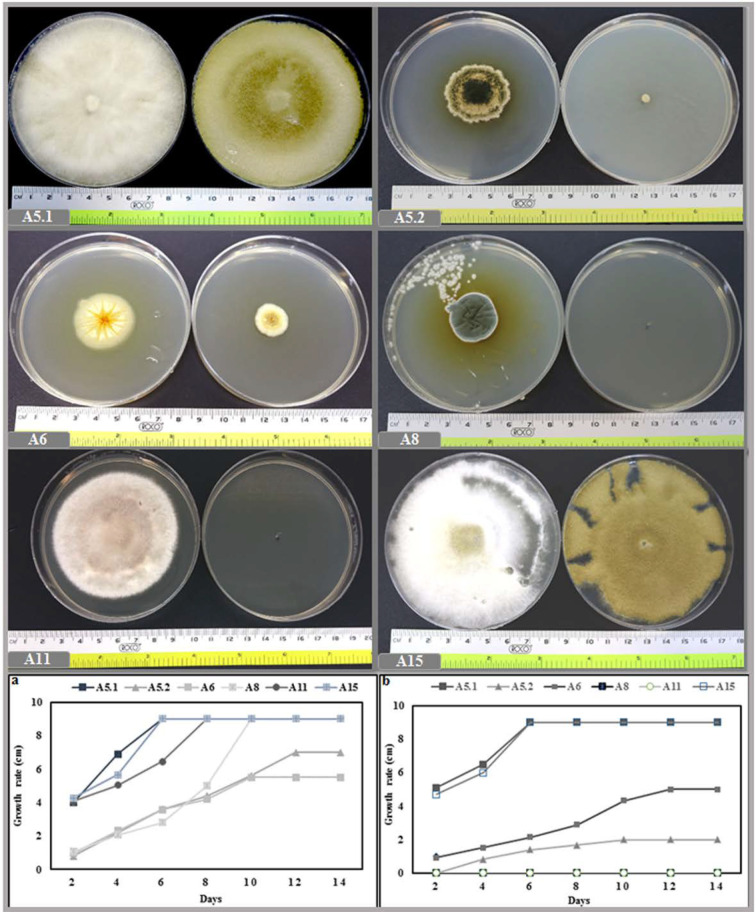
Effect of two incubation temperatures 27 °C (**a**) and 40 °C (**b**) on growth rate of the six isolated fungi. (**A5.1**: *Byssochlamys spectabilis*, **A5.2**: *Chaetomium globosum*, **A6***: Cephalotheca foveolata*, **A8**: *Penicillium melinii*, **A11**: *Alternaria tenuissima* and **A15**: *Nigrospora chinensis*).

The growth rate was investigated by measuring the diameter of the strains at daily intervals during the incubation period. The result of the incubation at 27 °C showed that the six isolates could be classified into fast growing strains (A5.1 and A15), in which the colonies reached the edge the petri dish quickly (6 days after inoculation). The growth rates of A8 and A11 were classified as moderate, since these two strains required more time to cover the whole surface of the medium (10 and 8 days, respectively). For the slow growing fungi (A5.2 and A6), the maximum diameters of growth achieved were, respectively, 7 and 5.5 cm, after 14 days of incubation. In addition, at 40 °C the A5.1 and A15 strains grew normally and reached the edge of the petri dish within 6 days. However, A5.2 and A6 grew slowly, while A8 and A11 were totally inhibited (Figure 2). At a temperature of 50 °C, the growth of A5.2 and A6 was stopped, whereas a small amount of radial growth of A5.1 and A15 was registered (Supplementary Figure S3).

**Figure 3:**
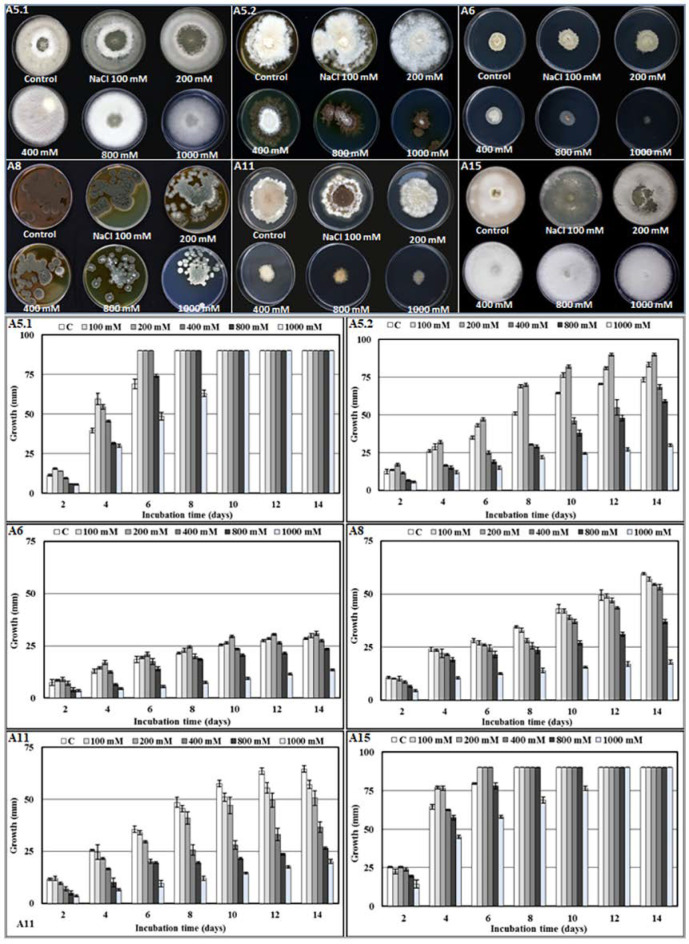
Morphology and growth rate of the six isolated strains (**A5.1**, **A5.2**, **A6**, **A8**, **A11**, and **A15**) incubated at different NaCl concentrations (100, 200, 400, 800, and 1000 mm) during 14 days at 27 °C. (**A5.1**: *Byssochlamys spectabilis*; **A5.2**: *Chaetomium globosum*; **A6***: Cephalotheca foveolata*; **A8**: *Penicillium melinii*; **A11**: *Alternaria tenuissima*; and **A15**: *Nigrospora chinensis*).

The investigation into the effect of salinity on mycelial growth showed that, even though the six isolates were able to grow under 1 M NaCl, the strains displayed different growth characteristics under differing levels of salt. Interestingly, isolates A5.1 and A15 were more tolerant to NaCl; furthermore, under 100 mm and 200 mm NaCl, the two strains covered the entire surface of the agar more quickly than the controls. We noted also that strains A5.2 and A6 had the largest colony diameters at 200 mm NaCl. However, A8 and A11 were negatively affected by NaCl at all concentrations (Figure 3).

### 2.2. Evaluation of Fungi on Plant Growth Promoting Activity

#### 2.2.1. Evaluation of the Fungi Effect

**Figure 4:**
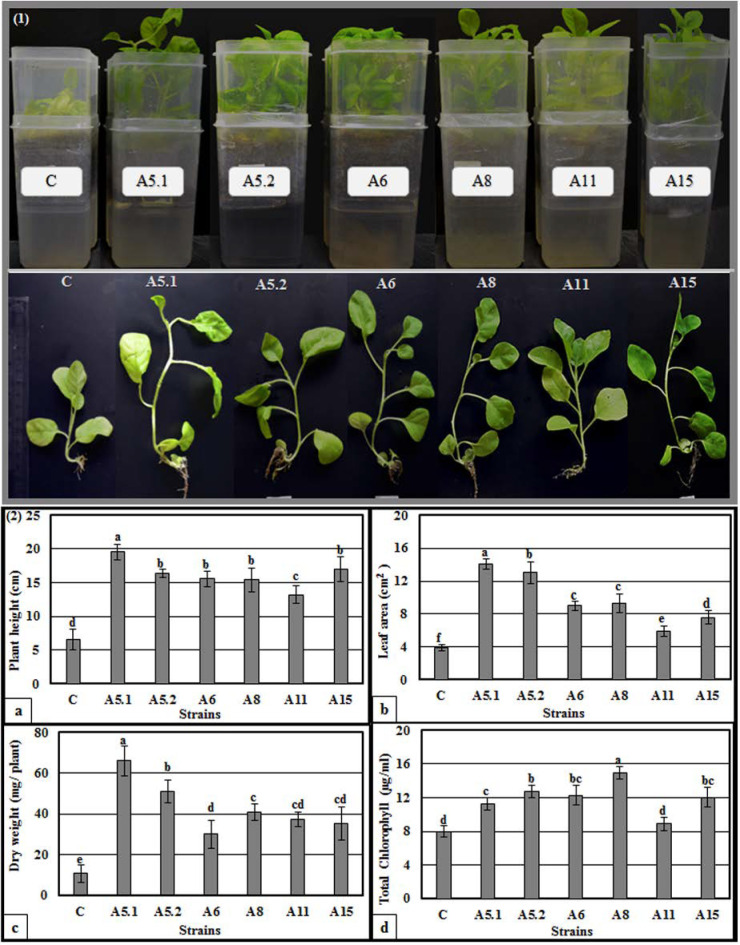
Direct effects of isolated fungi on promoting tobacco seedling growth in liquid MS medium. (**1**): Morphology of tobacco seedlings directly affected with six isolated fungi. For treated plants, MS medium was inoculated with five microliters of the isolated fungi two weeks after sowing. (**2**): Direct effects of isolated fungi on plant height (**a**), leaf area (**b**), plant dry weight (**c**), and total chlorophyll (**d**). Data are the means of five replicates ± standard deviation; different letters on bars represent the significant values according to Duncan’s test (*p* < 0.05). (A5.1: *Byssochlamys spectabilis*, A5.2: *Chaetomium globosum*, A6: *Cephalotheca foveolata*, A8: *Penicillium melinii*, A11: *Alternaria tenuissima* and A15: *Nigrospora chinensis*).

After germination, the bottoms of the plates were inoculated with liquid cultures of each fungus. Three weeks after fungal inoculation, the plants exhibited stimulated shoot and root growth, compared with the control plants (Supplementary Figure S4). Indeed, we observed a visible increase in shoot height, leaf number and surface area, root length, and number of the lateral roots, compared with non-treated plants. The evaluation of growth promotion was then performed in liquid MS medium inoculated with the fungi (Figure 4). All of the tested fungi were found to significantly increase plant height, leaf area, dry weight, and total chlorophyll content, compared with the controls. Importantly, plants treated with A5.1 showed the highest values for plant height, leaf area, and dry weight (19.6 cm, 14.12 cm^2^, and 66 mg, respectively; (Figure 4(2a–c)). The highest value of total chlorophyll was found in plants treated with strain A8 (14.9 µg/mL) (Figure 4(2d)). Based on these results, the CFFs of the six strains were selected to study their effects on plant growth promoting activities.

#### 2.2.2. Evaluation of the CFFs Effect

**Figure 5:**
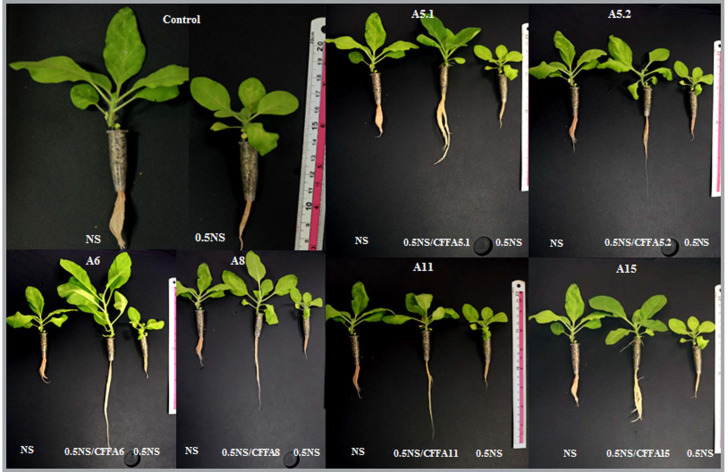
Phenotypes of tobacco seedlings grown in nutrients solution (NS) or (0.5NS) supplied or not with fungal cell-free filtrates (CFF 1/50). (**A5.1**: *Byssochlamys spectabilis*; **A5.2**: *Chaetomium globosum*; **A6***: Cephalotheca foveolata*; **A8**: *Penicillium melinii*; **A11**: *Alternaria tenuissima*; and **A15**: *Nigrospora chinensis*).

**Figure 6:**
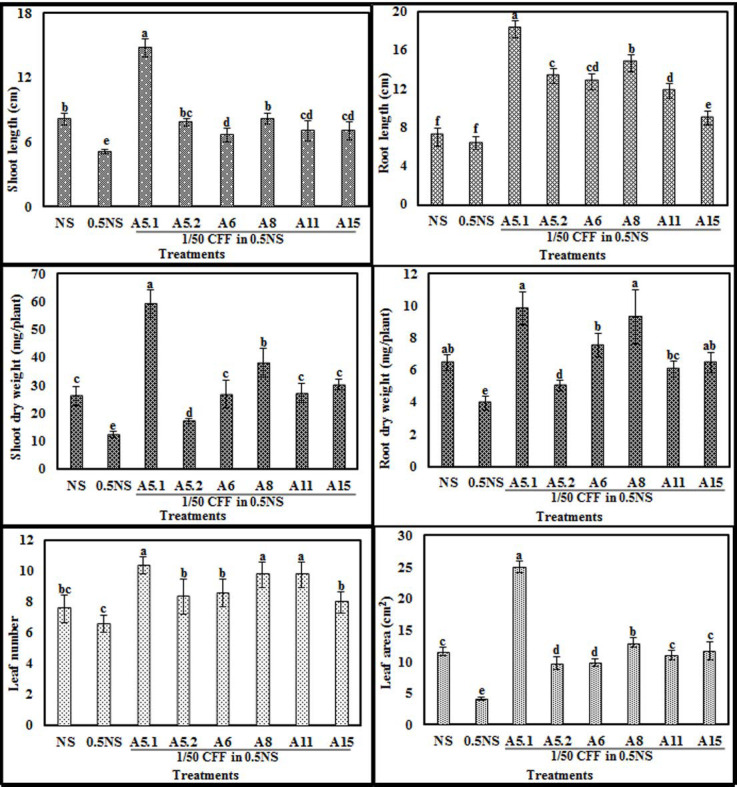
Effects of adding fungi cell-free filtrates CFFs (1/50) to half nutrient solution (0.5NS) compared to control full NS and 0.5NS on shoot length, root length, shoot dry weight, root dry weight, leaf number, and leaf area of tobacco seedlings grown in a hydroponic system. Values represent means and standard deviations (n = 5), where samples labeled with different letters are significant at *p* < 0.05.

The biostimulant activities of the CFFs on plant phenotype are presented in the Figure 5 and (Supplementary Figure S5). The results showed that adding the CFFs from all fungal strains to 0.5NS at a concentration of 1/50 significantly increased shoot length, root length, shoot dry weight, root dry weight, leaf number, and leaf area, compared with those of untreated plants grown either in NS or 0.5NS (Figure 6). The CFF of A5.1 added to 0.5NS induced the best performance in enhancing plant vigor when compared to either NS or 0.5NS. Indeed, the CFF of A5.1 significantly increased (at least doubled) the values of all tested parameters including shoot and root length, dry weight, leaf number, and leaf area. The CFFs of all isolated fungi improved not only plant vigor but also nutrient-use efficiency, since the plants cultivated in 0.5NS containing CFF (1/50) exhibited the best growth performance (Figure 6). These findings support the suggestion that these isolated fungi could be used as cost-effective biostimulants and decrease the use of chemical fertilizers.

### 2.3. Presence of Auxin in Culture Filtrates

**Figure 7:**
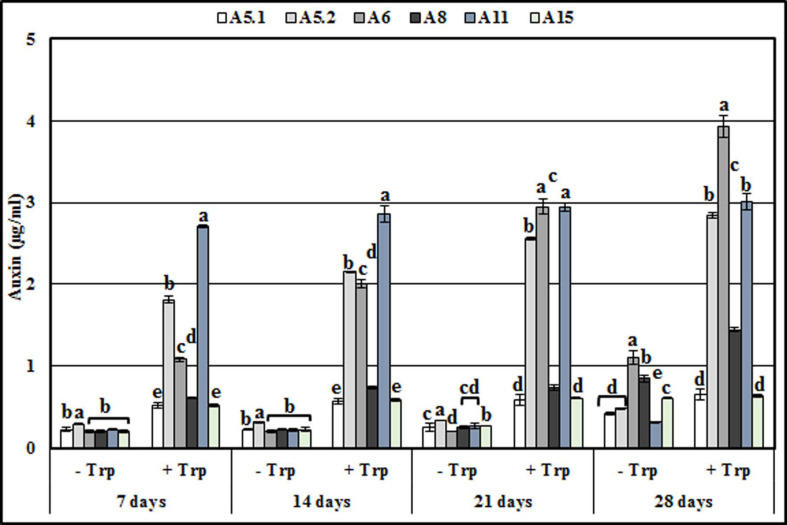
Production of IAA by the six isolated fungi in the presence or absence of L-Tryptophan during 7, 14, 21 and 28 days of incubation. Values represent means and standard deviations (n = 5), where samples labelled with different letters are significant at *p* < 0.05.

The present study showed that the six isolates were capable of synthesizing auxin during growth in the culture medium (Figure 7). In the absence of L-tryptophan and during up to 21 days of incubation, the six strains produced almost the same amount of auxin as each other, while after 28 days, A6 and A8 showed a maximum auxin production (1.1 ppm and 0.84 ppm, respectively). The addition of L-tryptophan into the culture media substantially increased the auxin production of the six strains. Furthermore, for the period up to 21 days of incubation, the strains could be divided into, low auxin producers (A5.1, A8, and A15) and high IAA producers (A5.2, A6, and A11). However, after 28 days, auxin production was higher in A5.2, A6, and A11 (2.83, 3.9, and 2.68 ppm, respectively), moderate in A8 (1.44 ppm), and lower in A5.1 and A15 (0.65 and 0.63 ppm, respectively).

### 2.4. Effect of Fungal Culture Filtrates on the Expression Profiles of Growth Related Genes

**Figure 8:**
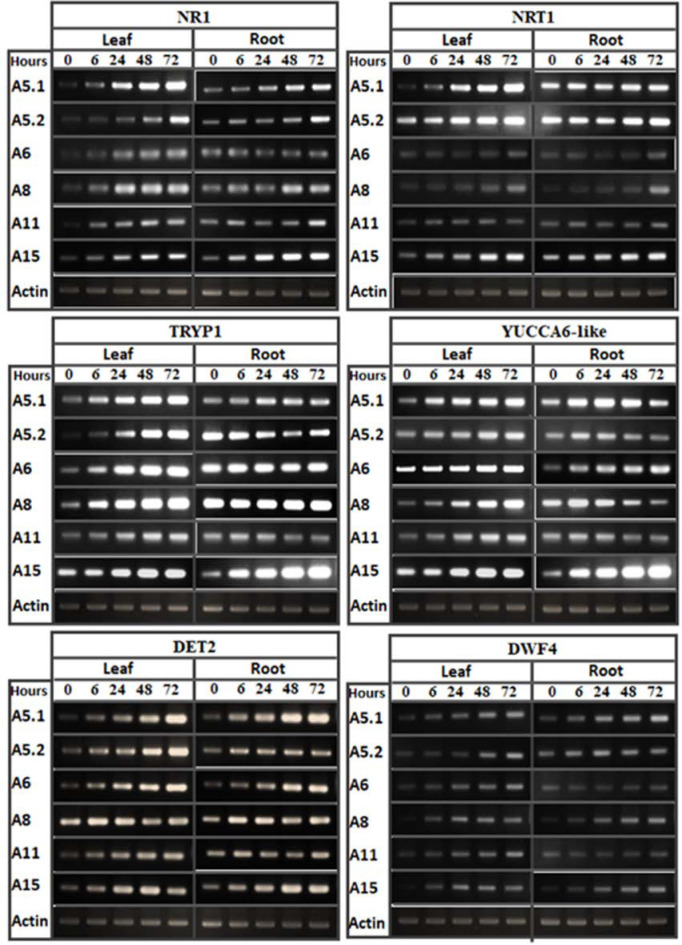
RT-PCR analysis showing the effect of culture filtrates on gene expression implicated in nitrogen-metabolism (NR1, NRT1), auxin biosynthesis (Tryp1, YUCCA6-like) and brassinosteroid biosynthesis (DET2, DWF4). The RT-PCR was performed in leaf and root at 0, 6, 24, 48, and 72 h of filtrate application. Actin gene was used as reference.

In tobacco leaves and roots treated with a 1/50 dilution of CFF (at 0, 6, 24, 48, and 72 h post-treatment), RT-PCR analysis was used to evaluate the accumulation of transcripts for six genes related to brassinosteroid hormone biosynthesis (*DET2* and *DWF4*), auxin biosynthesis (*YUCCA6-like* and *Tryp1*), and nitrogen use efficiency (*NR1* and *NRT1*). The results showed that the brassinosteroid and auxin biosynthesis genes were up-upregulated in both leaf and root after CFF application (Figure 8). However, the accumulation of transcripts differed kinetically among the genes and treatments. Despite differences between strains and plant organs in levels of abundance of *NR1* and *NRT1* transcripts, these two genes were in fact induced after the cell-free filtrate application. Based on these results, it was concluded that the induction of *DET2*, *DWF4*, *YUCCA6-like*, and *Tryp1* could be considered as one explanation for the stimulation of root and shoot vigor due to increases in brassinosteroids and auxins. This could be in addition to the beneficial effect of the IAA detected in the CFs. However, the increase in transcript accumulation of the nitrogen use efficiency genes probably explains the stimulation of plant vigor in 0.5NS containing 1/50 CFF.

## 3. Discussion

The main goals of smart agriculture are to assure the yield and the quality of crops by developing new organic and sustainable production systems. For this, reducing fertilizers inputs and saving water consumption are alternative approaches that should be persuaded in the development of hydroponics growing systems. Biostimulants are used in combination with conventional fertilizers to improve nutrient use efficiency or crops quality [21]. The PGPF are heterogeneous group of non-pathogenic soil-borne filamentous fungi associated with mediating the stimulation of plant growth and health [22]. Based on previous reports, PGPF differ from one another in habitat, taxonomy, physiology, and manner of interaction with plants [23]. The majority of PGPF belong primarily to the phyla: Ascomycota (*Aspergillus*, *Aureobasidium*, *Chaetomium*, *Cladosporium*, *Colletotrichum*, *Exophiala*, *Penicillium*, *Trichoderma*, *Fusarium*, *Gliocladium*, *Phoma*, *Phomopsis*, *Purpureocillium*, and *Talaromyces*), whereas a few of them belong to the Basidiomycota (*Limonomyces*, *Rhodotorula*, and *Rhizoctonia*) and Zygomycota (*Mucor* and *Rhizopus*) [24]. Reference [25] reported that PGPF represent around 44% of isolates from rhizospheres.

In this study, six soil fungi were isolated from the rhizosphere of the extremophile plant *Aeluropus littoralis*, living in salty soil. These fungi were identified using ITS sequences as: *B. spectabilis* (A5.1), *C. globosum* (A5.2), *C. foveolata* (A6), *P. melinii* (A8), *A. tenuissima* (A11), and *N. chinensis* (A15). These ITS markers have been used widely to identify new strains [26,27,28]. The ITS regions are important molecular targets for species identification due to the amount of sequence variation that exists within them and the speed at which these sequences evolve [29,30]. Two fungal species, A5.1 and A15, were shown to be tolerant to high temperatures (up to 40 °C) and high levels of NaCl (up to 1000 mm). The other four fungi showed a moderate (A5.2 and A6) or weak tolerance (A8 and A11) to high temperatures and NaCl. At 50 °C all of the fungi died, except for A5.1 and A15, grew very slowly. It was reported that *Byssochlamys* sp. strains are often heat resistant [31]. In addition, it was demonstrated that sexual reproduction is the main cause of heat resistance in the fungus *B. spectabilis*, which is the teleomorph (sexual form) of *Paecilomyces variotii* [32]. To our knowledge, our results show for the first time that *N. chinensis* is a heat-tolerant fungus and that *B. spectabilis* and *N. chinensis* can grow normally in the presence of 1000 mm NaCl. In another study, it was found that two Trichoderma isolates TaDORS3 and TaDOR693 had maximum colony growth at 1 M NaCl concentration indicating their high osmotolerance level [33]. IAA and gibberellin are important secondary metabolites produced commercially from fungi for the agricultural and horticultural industries [34]. Many microorganisms secrete signaling compounds in the rhizosphere as phytohormones that can stimulate plant growth [10,35,36]. In the present study, the six isolated fungi produced the auxin, but the concentrations became more significant when the culture medium was supplemented with L-tryptophan. This confirms the result reported by [37], who noted that the IAA concentration was higher in media supplemented with tryptophan compared to control media. Moreover, reference [16] described fungal endophytes as producers of IAA in their cultures.

After isolation and identification, the six fungal strains were screened for their potential as PGPF. Our results showed that the positive effect of applying the six fungi did not require direct contact between the fungus and plant roots. This can explain the significant growth improvement of tobacco seedling following the application of the used fungi CFFs. Interestingly, the addition of the CFFs at a dilution of 1/50 in the half strength nutritive solution (0.5NS) resulted in same growth rate or better vigor of plants, especially with strains *B. spectabilis* (A5.1) and *P. melinii* (A8) (Figure 6). It was reported previously that in a field experiment, *B. spectabilis* caused an increase of about 42% in the herbage (*Ornithopus compressus*) yield, compared with the controls [38]. Recently, it was demonstrated that high values for percentage germination, seedling vigor, and root and shoot length were obtained following the direct application of *P. variotii* on tomato and pepper. This fungus *P. variotii* is the asexual state of *B. spectabilis* [39]. The results reported in this study concerning strain A5.2 are the same as shown by [40] for *Chaetomium globosum* D38C, which can stimulate plant growth and secondary metabolism of *Salvia miltiorrhiza* seedlings. *Cephalotheca foveolata* (A6) was identified as a new species of the genus *Cephalotheca* with a plant growth promoting activity. Another species, *C. sulfurea*, isolated from the roots of soybean was earlier reported to promote increases in shoot length [41]. In this work, a novel species, A8 (*P. melinii*), was shown to increase plant vigor. The plant growth promotion and stress mitigation effects of *Penicillium* sp. have also been demonstrated on sesame, cucumber, and maize plants [9,42,43]. The fungus *A. tenuissima* (A11), which is known as the main cause of leaf spot disease in many plants, was described for the first time in this study as a member of the PGPF. Finally, fungal species from the genus *Nigrospora* have been reported to have antifungal activity [44,45]. In this study, strain A15 (*N. chinensis*) was shown for the first time to have plant-growth promoting activity.

The results from our study and from those reported earlier show clearly that PGPF are classified as biostimulants. The use of this kind of biostimulant is one of the strategies for achieving sustainable agriculture [46]. The main reported benefits of PGPF include increased seed germination, improved plant vigor, better root architecture and morphogenesis, more shoot elongation, higher yield, improved photosynthetic efficiency, and increased flowering [24]. It has been demonstrated that some PGPF strains enhance plant growth through the secretion of plant phytohormones and volatiles [47,48]. Plant growth promotion by PGPF may also arise from enhanced nutrient availability, amelioration of abiotic stresses, and antagonism to phytopathogens [27,49]. PGPF most likely stimulates plant growth through one or more of these diverse mechanisms.

In order to increase the advantages of hydroponic closed system, it is important to find solutions to decrease the chemical fertilizers inputs without affecting plant growth. In addition, it is crucial to solve the increasing of the solution salinity during the plant cycle. The results reported in this work demonstrated that the addition of CFFs to the 0.5 NS can ensure a growth of plants same or better than those cultivated in full NS. The presence of IAA in the fungal culture could explain the growth stimulation. In addition, it was hypothesized that the filtrates could contain bioactive compounds or secondary metabolites, which may induce the expression of some genes implicated in growth regulator biosynthesis and/or nutrient-use pathways in plants. This could be explained in part by the upregulation of the genes involved in brassinosteroid (*DET2*, *DWF4*), auxin biosynthesis (*YUCCA6-like*, *Tryp1*), nitrogen use efficiency (*NR, NRT1*) (Figure 8). The plant growth promoting effect of CFFs was previously tested morphologically and physiologically [15,16,26,50]. However, the molecular mechanisms behind the addition of cell-free CFFs have not been reported previously. Some studies have analyzed plant molecular changes that occur under the direct effect of certain plant-beneficial fungi. It was found that *Piriformospora indica*, an endophytic fungus, promotes the growth of *Arabidopsis* and tobacco seedlings and enhances the expression of genes encoding nitrate reductase [51]. It was also noted that the endophytic fungus *C. cupreum* positively regulates the expression of some genes involved in auxin biosynthesis and metabolism in *Eucalyptus globulus* [52]. The modification of tomato root architecture is attributed to ethylene/indole-3-acetic acid signaling triggered by a strain of *Trichoderma* [53]. Similarly, in *Arabidopsis*, inoculation with *Trichoderma virens* increases the transcript accumulation of auxin genes [54]. It was also observed that *P. indica* significantly stimulates the expression of auxin-responsive reporter genes both in lateral root primordia and the root elongation zone of *Arabidopsis* within one day of inoculation [55].

## 4. Materials and Methods

In the present study, we isolated and identified six fungi strains from the rhizosphere of a halophyte grass *Aeluropus littoralis*. Their biostimulant activities were evaluated in the model tobacco seedlings grown in vitro or in hydroponic system. Moreover, the expression profiles of some genes involved in plant growth promotion were investigated.

### 4.1. Isolation of Fungi

Fungi isolation was performed following the method described by [56] with few modifications. The soil surrounding the roots of the halophyte plant *Aeluropus littoralis* (living in salty soil at Salboukh, located north of Riyadh, Saudi Arabia 25°04′48.6″ N 46°20′27.7″ E) was collected in a falcon tube by means of a sterilized spatula and taken to the laboratory. The soil was sieved and transferred into new sterile falcon tube containing autoclaved water. Thereafter, 100 µL from each tube were plated onto potato dextrose agar (PDA) (Scharlau), and the plates were then incubated at 27 °C for 5 days. When the mycelia emerged, single colonies of fungi were picked from the plates and each was transferred to a new PDA plate. Colonies were purified by repeated sub-culturing on PDA plates. In order to suppress bacterial growth, 100 mg L^−1^ of ampicillin was added to the PDA. The purified isolates were stored in 30% glycerol at −80 °C for further study.

### 4.2. Microscopic and Molecular Characterization

For microscopic observations, fungi were grown directly on glass slides. Each fungal colony was inoculated into a block of PDA (1 cm^2^) and placed on a sterile microscope slide, covered with a slip, then transferred to a sterile petri plate and incubated for 72 h at 27 °C. Thereafter, the cover slip was removed gently and placed on a new slide with a drop of lactophenol cotton blue. The slides were observed and photographed using a Nikon condenser microscope. Six isolates were identified according to their morphological characteristics.

Five-day-old mycelia were collected from a PDA plate and ground to a fine powder with a pestle and mortar using liquid nitrogen. Genomic DNA was extracted using a DNeasy Plant Mini Kit (Qiagen), according to the manufacturer’s instructions. The isolated DNA was quantified using a Nanodrop 8000 spectrophotometer (Thermo Scientific) and then used for PCR amplification of the internal transcribed spacer (ITS) regions. The ITS region was amplified using the universal primers ITS1 (forward) and ITS4 (reverse), as described by [57]. The PCR amplification was carried out in a 30 μL reaction volume containing 6 μL of PCR Master Mix 5X (Thermo Scientific), 0.5 μL of each primer, 4 μL of fungal genomic DNA, and 19 μL of water. The PCR reaction was performed in an Applied Biosystems Thermal Cycler, using the following conditions: one cycle at 94 °C for 5 min; 30 cycles at 94 °C for 1 min, annealing at 55 °C for 1 min, extension at 72 °C for 1 min; and a final extension step of 72 °C for 5 min. After running the amplified fragments on a 1.5% agarose gel, the PCR product was purified and cloned into pGEM^®^-T Easy vectors (Promega). All resulting DNA plasmids were sent to Macrogen Inc. (Seoul, Korea) for sequencing. The obtained sequences were subjected to a BLAST search of the NCBI databases to identify the isolated fungi.

### 4.3. Effect of Temperature and NaCl on Fungal Growth

To assess the effect of temperature on mycelial growth, the six isolates were inoculated on PDA and incubated at 27 °C, 40 °C, and 50 °C. The colony diameters were measured every 2 days during two weeks of growth and the mycelium was photographed one week after inoculation.

The effect of salinity on fungal growth was tested on Murashige and Skoog (MS) medium (sigma) at five NaCl concentrations (100, 200, 400, 800, and 1000 mm) with three replicates. The strains were subcultured at the center of 9 cm plastic petri dishes containing 20 mL medium and incubated at 27 °C. The mycelial diameter was registered every 2 days during 2 weeks of incubation.

### 4.4. Evaluation of Fungi on Plant Growth Promoting Activity

#### 4.4.1. Evaluation of the Fungi Effect

##### In Solid MS Medium

As a first step, isolated fungi were screened for their ability to promote plant growth on MS medium [58]. Wild type tobacco seeds were sterilized by immersion in 50% commercial Clorox for 15 min, washed vigorously with a sterile distilled H_2_O. Then, five seeds were germinated in each square petri plate (15 × 15 cm) containing solid MS medium and vertically incubated in the growth chamber. Ten days after sowing, 5 µL each of the six different fungi were inoculated onto the bottom of each petri dish. Control seedlings were maintained on MS medium without fungal inoculation. For each treatment five plates were used and the whole experiment was repeated three times. After three weeks, the plates were photographed.

##### In Liquid MS Medium

Screening was performed in two vertically assembled plastic boxes containing liquid MS medium. The uppermost box containing 15 small holes was filled with sterilized vermiculite (ArabianVermiculite Industries) and fixed on the box below containing sterilized MS liquid media. Then, five sterilized tobacco seeds were germinated in the vermiculite and five boxes were used for each condition. One week later, treatments were performed by inoculating 5 µL of each of the isolated fungi in the MS medium. Thirty days after inoculation, plants were harvested and data were recorded for plant height, leaf area, dry weight, and total chlorophyll content.

#### 4.4.2. Evaluation of the CFFs Effect

##### Preparing the Cell-Free Culture Filtrate (CFF)

A small 5 mm disc was picked from a 7-day-old culture on solid PDA medium of each purified strain and inoculated into Erlenmeyer flasks (250 mL) containing 100 mL of MS medium. The flasks were incubated at 27 °C at a shaking speed of 150 rpm for 30 days. The culture cell-free filtrate (CFF) was obtained using vacuum filtration unit (0.2 µm) and stored at 4 °C for further use.

##### Adding the Fungal Cell-Free Culture Filtrates (CFF) to Nutrient Solution

Wild-type tobacco seeds were sterilized by immersion in 50% commercial Clorox for 15 min, then washed with distilled water three times and placed in sterile 1.5 mL Eppendorf tubes (perforated from the bottom) containing sterile vermiculite. The tubes were fixed in small plastic cups and kept in an autoclaved box for germination to occur. Ten days post-sowing, the seedlings were transplanted into a hydroponic growing system containing full (NS) and half (0.5NS) of nutrient solution (NS was prepared by mixing in one liter: 600 mg Chem-Gro^TM^ fertilizer 8-15-36, 600 mg calcium nitrate (CaNO_3_), 373 mg magnesium sulfate (MgSO_4_) and the pH is adjusted to 6.2). For treatments, one week after transplantation, the 0.5NS was supplemented with a 1/50 dilution of the fungal cell-free culture filtrates. The plants were grown under controlled conditions at a constant temperature 25 °C and a 16 h/8 h light/dark photoperiod. One-month post-inoculation, the growth parameters—such as shoot and root length, shoot and root dry weight, leaf number, and leaf area—were measured using five replications and each one is composed of five plants for tested conditions.

### 4.5. Total Chlorophyll Estimation

The total chlorophyll was extracted in the acetone 80%, and the content was calculated according to the equation of [59]: Total chlorophyll (μg/mL) = 20.2 (A645) + 8.02 (A663). The A663 and A645 represent absorbance values read at 663 and 645 nm wavelengths, respectively.

### 4.6. Estimation of Auxin Concentration in Culture Filtrate

The production of auxin was measured according to the methods described by [60]. In brief, the six strains of fungi were inoculated in MS medium with or without of L-tryptophan (1 mg mL^−1^) and incubated at 27 °C for 7, 14, 24, and 28 days. After each period of incubation, the culture was centrifuged at 10,000 rpm for 15 min. Then, 1 mL of the supernatant was mixed with 1 mL of Salkowski reagent (1 mL 0.5 M FeCl_3_, 30 mL concentrated H_2_SO_4_, and 50 mL distilled H_2_O) and allowed to react in dark for 20 min at room temperature. The optical density was read at 530 nm and the amount of IAA produced was calculated by comparing with the standard IAA curve.

### 4.7. Analysis of Gene Expression

The effect of the CFFs on the expression of genes implicated in nitrogen metabolism (*NR1* and *NRT1*), auxin biosynthesis (*Tryp1* and *YUCCA6-like*), and brassinosteroid biosynthesis (*DET2* and *DWF4*) was tested at 0, 6, 24, 48, and 72 h after applying the CFFs to the growing media using semi-quantitative RT-PCR. Total RNA was extracted using a Qiagen RNAeasy Plant mini kit, following the manufacturer’s instructions. After quantification, total RNA was treated with DNaseI (RQ1, Promega, USA) to remove DNA contamination. For cDNA synthesis, 5 μg of total RNA were reverse transcribed with random hexamer primers using SuperScript™ III reverse transcriptase (Invitrogen) and oligo-(dT18), as described in the manufacturer’s instructions. This cDNA was used for PCR reactions performed on an Applied Biosystems Thermal Cycler as follows: 95 °C for 3 min, followed by 40 cycles of 95 °C for 20 s, 60 °C for 30 s, and 72 °C for 1 min. The actin genes (380 bp, *ACT-F* and *ACT-R*) were selected as references in the analysis of gene expression under normal growing conditions and after the application of the CFs. The primers used for gene amplification were designed using Primer3 software (http://bioinfo.ut.ee/primer3-0.4.0 (accessed on 4 March 2021)) (Supplementary Table S1). For each biological sample per condition, the semi-quantitative PCR was performed using three technical replicates and the products were analyzed on a 1.5% agarose gel.

### 4.8. Statistical Analysis

One-way ANOVA of SPSS software was performed and then data were analyzed with Duncan’s test. Each value is the mean of five replicates for all investigated growth parameters. Different letters were used to indicate means that differ significantly at *p* ≤ 0.05.

## 5. Conclusions

The results of this study show that all of the six fungal species isolated from the rhizosphere of a halophyte grass, *Aeluropus littoralis*, exert plant promoting activities of different intensities, either directly or indirectly. When the cell-free culture filtrates (CFFs) were added to 0.5NS in a closed hydroponic system, the greatest effects on tobacco seedlings were seen following the additions of *B. spectabilis* (A5.1) and *P. melinii* (A8). To our knowledge, this is the first demonstration of plant-growth promotion associated with *C. foveolate* (A6), *P. melinii* (A8), *A. tenuissima* (A11), and *N. chinensis* (A15). In addition, we proved that the CFF of all of isolated fungi when added to 0.5NS can decrease the chemical inputs with a biomass production equal or significantly better than that produced in full NS. In an attempt to explain the mechanism behind the growth stimulation, the transcript levels of six genes involved in nitrogen-metabolism (*NR1* and *NRT1*), auxin biosynthesis (*Tryp1* and *YUCCA6-like*) and brassinosteroid biosynthesis (*DET2* and *DWF4*) showed an upregulation either in roots or leaves following treatment of the plants with CFFs. Future work is needed to further study the mechanisms that explain the plant growth stimulation activity especially with the fungus A5.1 and A8. This may include investigation differential genes’ expression using RNAseq technology. In addition, we will investigate the ability of CFFs in mitigating abiotic or biotic stresses.

## Supplementary Materials

The following are available online at https://www.mdpi.com/article/10.3390/plants10040784/s1, Figure S1 Colony morphology of the six isolated fungi grown on PDA (72 h at 27 °C) and their microscopic observation (40X); Figure S2 The genomic DNA (a) and the PCR amplification product (b) of the rDNA-ITS gene of the six isolated strains; Figure S3 Effect of 50 °C temperature on growth rate of the six isolated strains during 14 days of incubation; Figure S4 Effect of isolated fungi on tobacco seedling growth. Seeds of tobacco were cultivated on MS agar medium in square plates, 10 days after sowing; inoculation was performed in the bottom of plates. One month post sowing plates were photographed; Figure S5 Shoots and roots phenotypic variation of tobacco seedlings grown in nutrients solution NS or 0.5NS supplied or not with fungal cell-free filtrates (CFF 1/50). Table S1. The primers sequences used in the semi quantitative RT-PCR.
