# Supplementary material for: Harnessing the Rhizosphere of the Halophyte Grass Aeluropus littoralis for Halophilic Plant-Growth-Promoting Fungi and Evaluation of Their Biostimulant Activities
Source: Plants (Basel). 2021 Apr 16;10(4):784. doi: 10.3390/plants10040784 (PMC8073152; doi:10.3390/plants10040784)
Supplement: Supplementary file 1 [file plants-10-00784-s001.zip › plants-1151415-supplementary.new/table S1.pdf]

Table S1. The primers sequences used in the semi quantitative RT-PCR

Primer	Sequence
YUC6-F	GGTTTGCTTGGTGCTTCCAT
YUC6-R	CTTTTCCTCTTTCACCTTGTCGT
Tryp1-F	GCAGAGGCTTATGTCTATTCAAGG
Tryp1-R	ATCCAAGTTTCATTGCTGTAAAAAG
DET2-F	TTTGGTTCCTAGAGCTTGTGC
DET2-R	TCAAGGTAAAGCCAAATACAGTCC
DWF4-F	GCCTTTTGCCTTCCCTTACG
DWF4-R	CTGAGGTGGAGCCTAACGTC
NR1-F	ATGTCACAATACCTTGATTCTCTCC
NR1-R	AGTTATCCCTGTTCCACCTGC
NRT1-F	AGAGACTTGCTGAAATGGGGA
NRT1-R	GATGATGGAGTAACAAGCGCA
ACT-F	GTGCCCATTTACGAACGATA
ACT-R	GAAGACTCCATGCCGATCAT
