# Supplementary figures and images for: Harnessing the Rhizosphere of the Halophyte Grass Aeluropus littoralis for Halophilic Plant-Growth-Promoting Fungi and Evaluation of Their Biostimulant Activities
Source: Plants (Basel). 2021 Apr 16;10(4):784. doi: 10.3390/plants10040784 (PMC8073152; doi:10.3390/plants10040784)

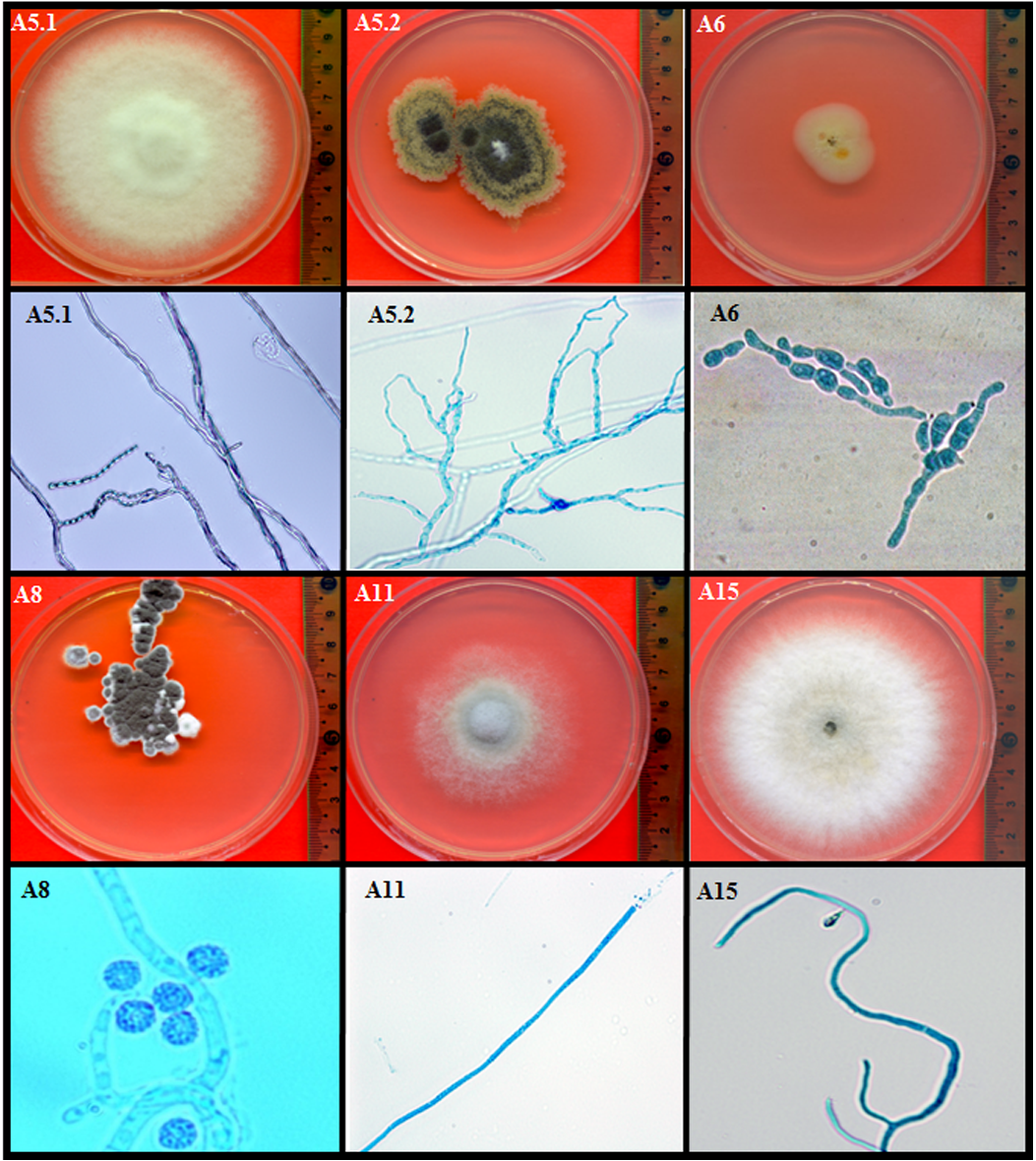

Supplement: Supplementary file 1 [file plants-10-00784-s001.zip › plants-1151415-supplementary.new/FigS1.tif]

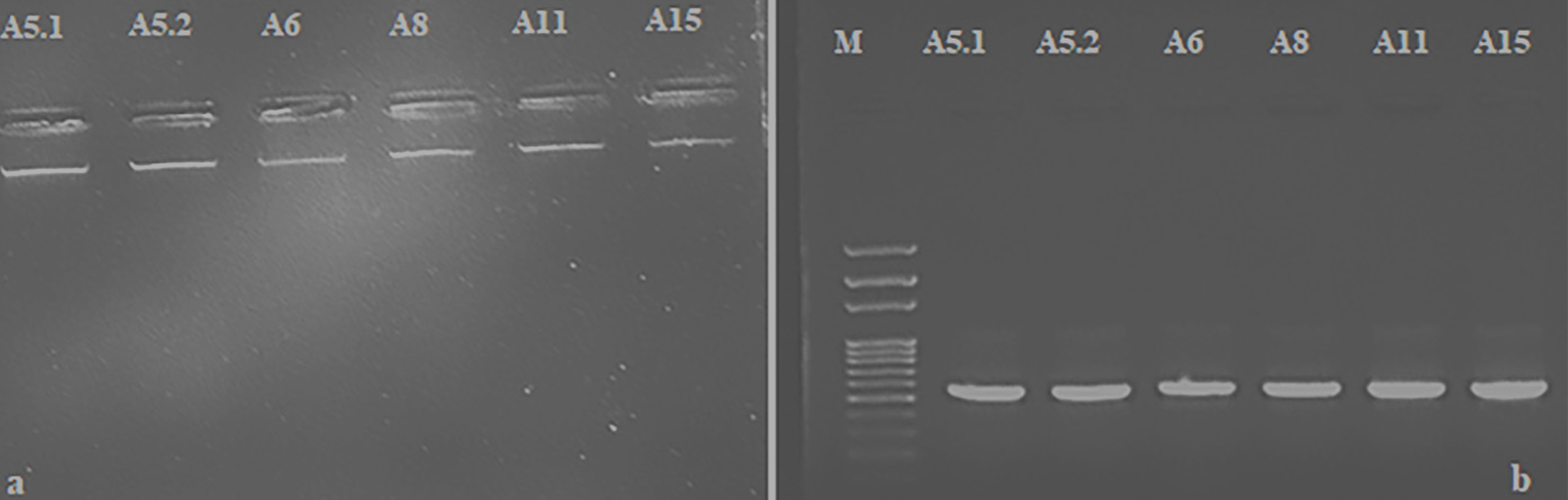

Supplement: Supplementary file 1 [file plants-10-00784-s001.zip › plants-1151415-supplementary.new/FigS2.tif]

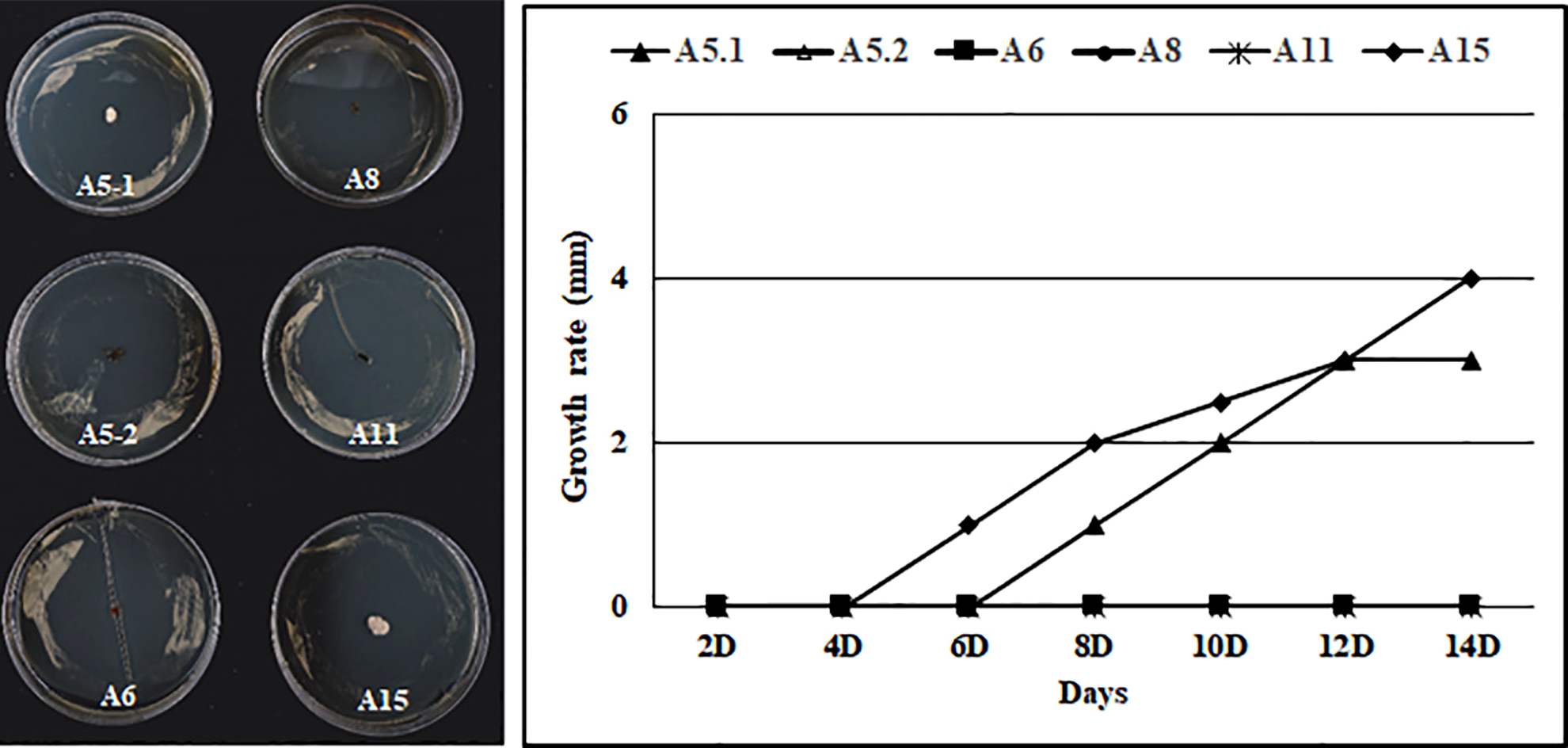

Supplement: Supplementary file 1 [file plants-10-00784-s001.zip › plants-1151415-supplementary.new/FigS3.tif]

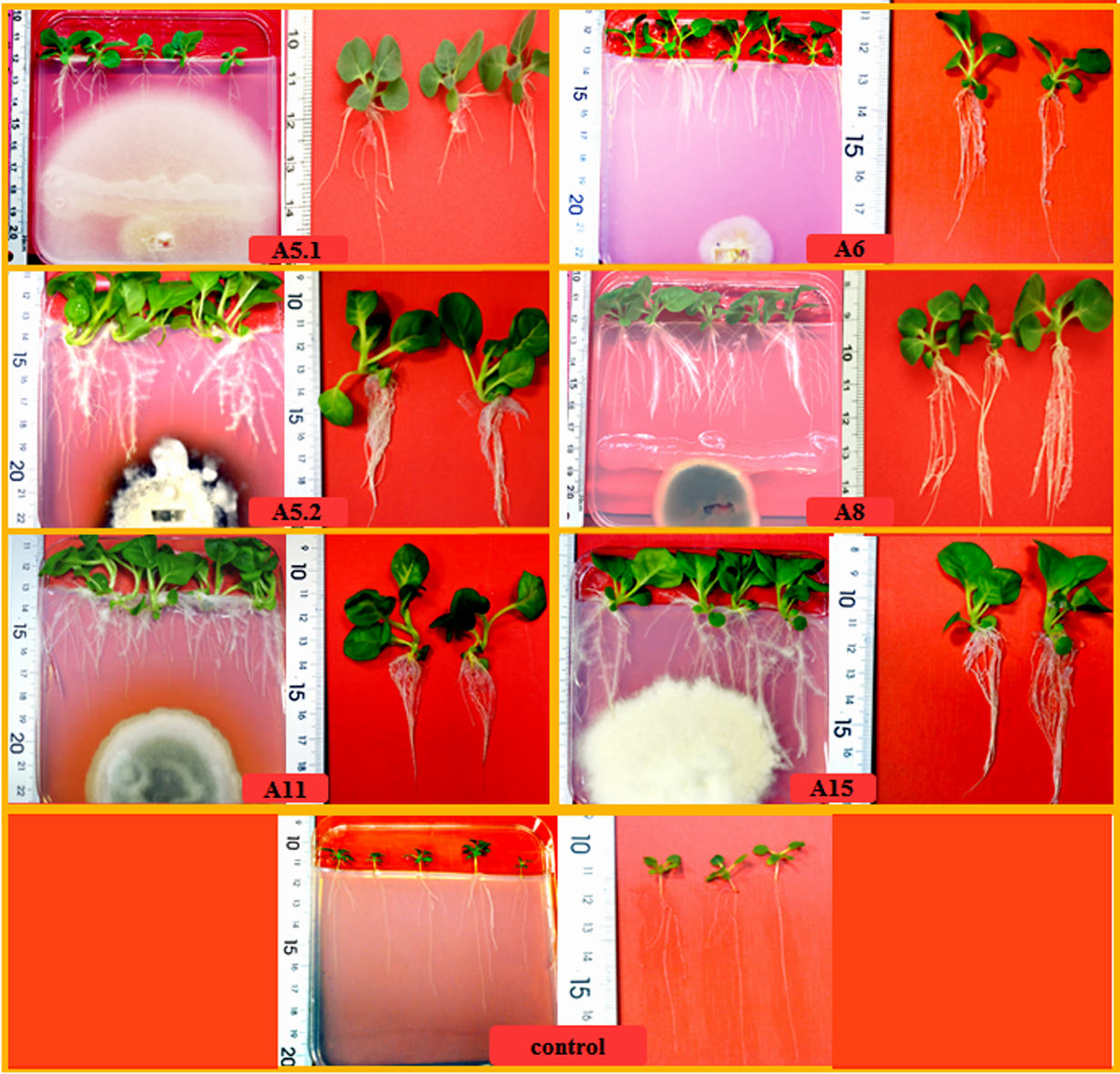

Supplement: Supplementary file 1 [file plants-10-00784-s001.zip › plants-1151415-supplementary.new/FigS4.tif]

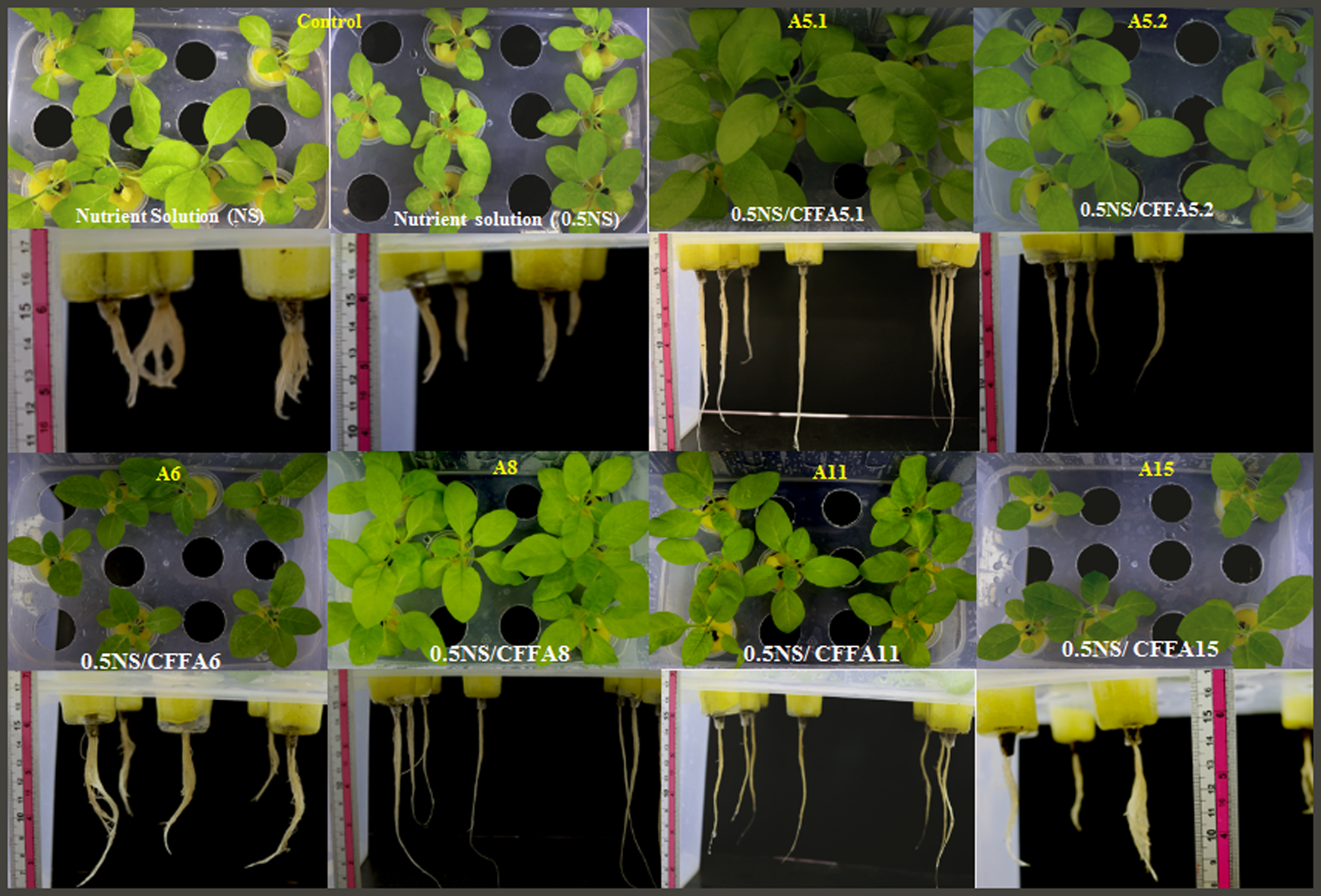

Supplement: Supplementary file 1 [file plants-10-00784-s001.zip › plants-1151415-supplementary.new/FigS5.tif]
